# Supplementary material for: High-resolution view of HIV-1 reverse transcriptase initiation complexes and inhibition by NNRTI drugs
Source: Nat Commun. 2021 May 4;12:2500. doi: 10.1038/s41467-021-22628-9 (PMC8096811; doi:10.1038/s41467-021-22628-9)
Supplement: Supplementary file 1 — Supplementary Information [file 41467_2021_22628_MOESM1_ESM.pdf]

Supplementary Information:

High-resolution view of HIV-1 Reverse Transcriptase initiation complexes and inhibition by NNRTI drugs

Betty Ha[1,2,7], Kevin P. Larsen[1,3,5,7], Jingji Zhang[1,7], Ziao Fu[4,6], Elizabeth Montabana[1], Lynnette N. Jackson[1], Dong-Hua Chen[1], Elisabetta Viani Puglisi[1,*]

[1] Department of Structural Biology, Stanford University School of Medicine, Stanford, CA. [2]Department of Molecular and Cellular Physiology, Stanford University School of Medicine, Stanford, CA. [3]Program in Biophysics, Stanford University, Stanford, CA. [4]Department of Chemistry and Molecular Biophysics, Columbia University, New York, NY. [5]Present address: University of California Berkeley, Department of Molecular and Cell Biology, Berkeley, CA. [6]Present address: Laboratory of Molecular Neurobiology and Biophysics, The Rockefeller University, Howard Hughes Medical Institute, New York, NY. [7]These authors contributed equally to this work: Betty Ha, Kevin P. Larsen, Jingji Zhang. *Correspondence: epuglisi@stanford.edu

Contents:

Supplementary Figure 1: miniRTIC purification and activity validation.
Supplementary Figure 2: Cryo-EM data processing and validation.
Supplementary Figure 3: Additional cryo-EM processing, local resolution estimates, and example density.
Supplementary Figure 4: RTIC cryo-EM density and modelling.
Supplementary Figure 5: miniRTIC protein–RNA contact analysis.
Supplementary Figure 6: NNRTI binding to the RTIC.

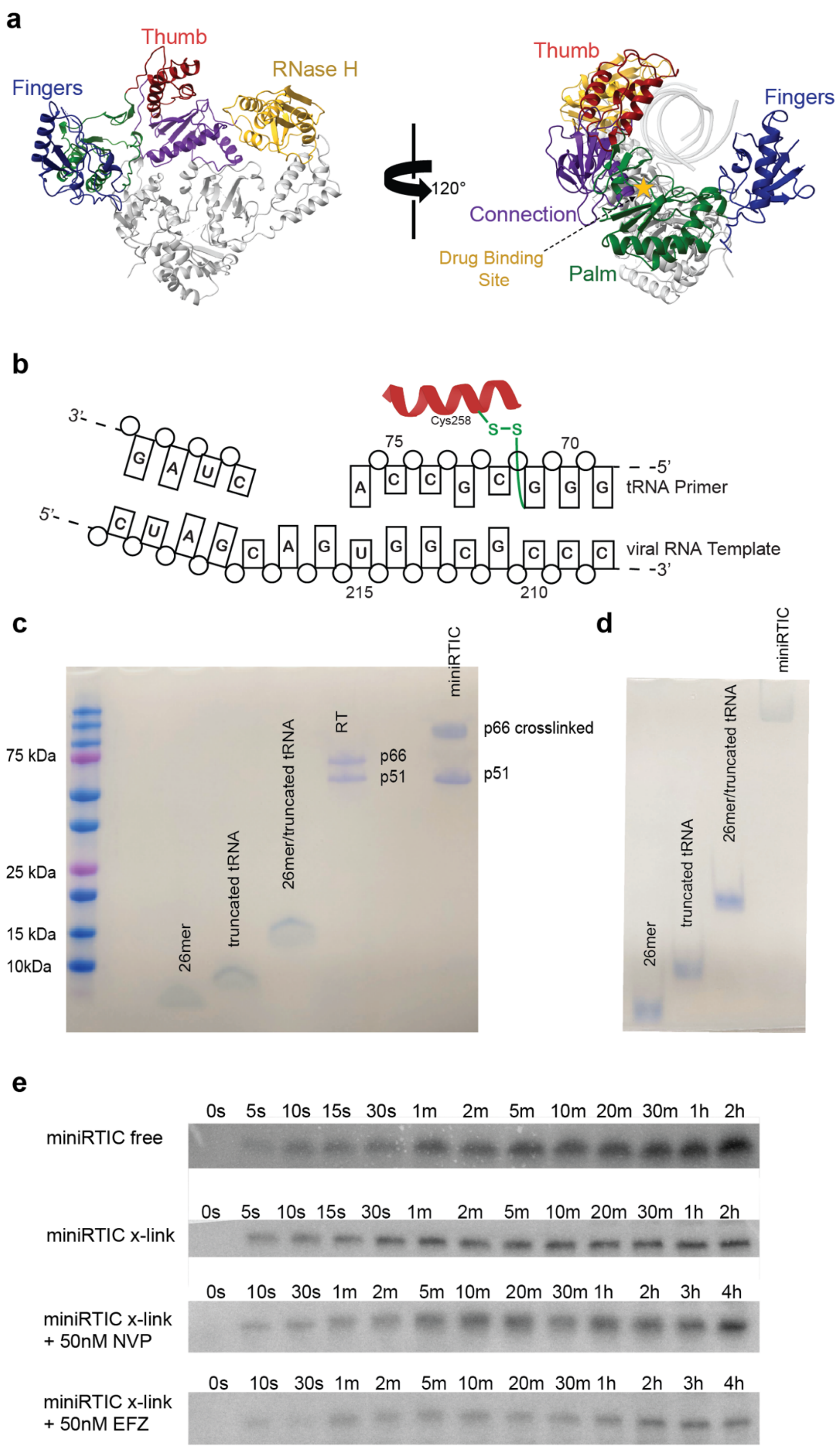
a
Thumb
RNase H
Fingers
120°
Thumb
Fingers
Connection
Drug Binding Site
Palm
b
Cys258
S–S
75
70
3'
5'
tRNA Primer
viral RNA Template
-3'
215
210
c
75 kDa
25 kDa
15 kDa
10kDa
26mer
truncated tRNA
26mer/truncated tRNA
RT
p66
p51
miniRTIC
p66 crosslinked
p51
d
26mer
truncated tRNA
26mer/truncated tRNA
miniRTIC
e
miniRTIC free
0s 5s 10s 15s 30s 1m 2m 5m 10m 20m 30m 1h 2h
miniRTIC x-link
0s 5s 10s 15s 30s 1m 2m 5m 10m 20m 30m 1h 2h
miniRTIC x-link + 50nM NVP
0s 10s 30s 1m 2m 5m 10m 20m 30m 1h 2h 3h 4h
miniRTIC x-link + 50nM EFZ
0s 10s 30s 1m 2m 5m 10m 20m 30m 1h 2h 3h 4h

Supplementary Fig. 1: miniRTIC purification and activity validation. (a) RT domain architecture. The p66 subunit is coloured by domain (Fingers = blue, Palm = green, Thumb = red, Connection = purple, RNase H = yellow) and the p51 subunit is grey. The NNRTI binding pocket is marked with a star. **(b)** Crosslinking scheme of the miniRTIC (Thumb αH=red, crosslink=green). The crosslinkable nt is placed at position 71. **(c)** Non-reducing SDS-PAGE of the vRNA (26mer), truncated tRNA, free vRNA–tRNA, reverse transcriptase (RT), and miniRTIC. The miniRTIC exhibits a shifted p66 subunit due to crosslinking with the vRNA–tRNA. **(d)** Native PAGE (20% polyacrylamide) of 26mer, truncated tRNA, vRNA–tRNA, and miniRTIC. The miniRTIC runs as a single band. Gel analysis in **c** and **d** was performed on all samples used in the manuscript ($n > 5$) and was consistently reproducible. **(e)** Representative autoradiograph image of incorporation of dCTP over time in free RT+vRNA–tRNA (miniRTIC free), crosslinked miniRTIC (miniRTIC x-link), miniRTIC x-link in the presence of 50 nM nevirapine and miniRTIC x-link in the presence of 50 nM efavirenz. Results are quantified and plotted in Fig. 1d.

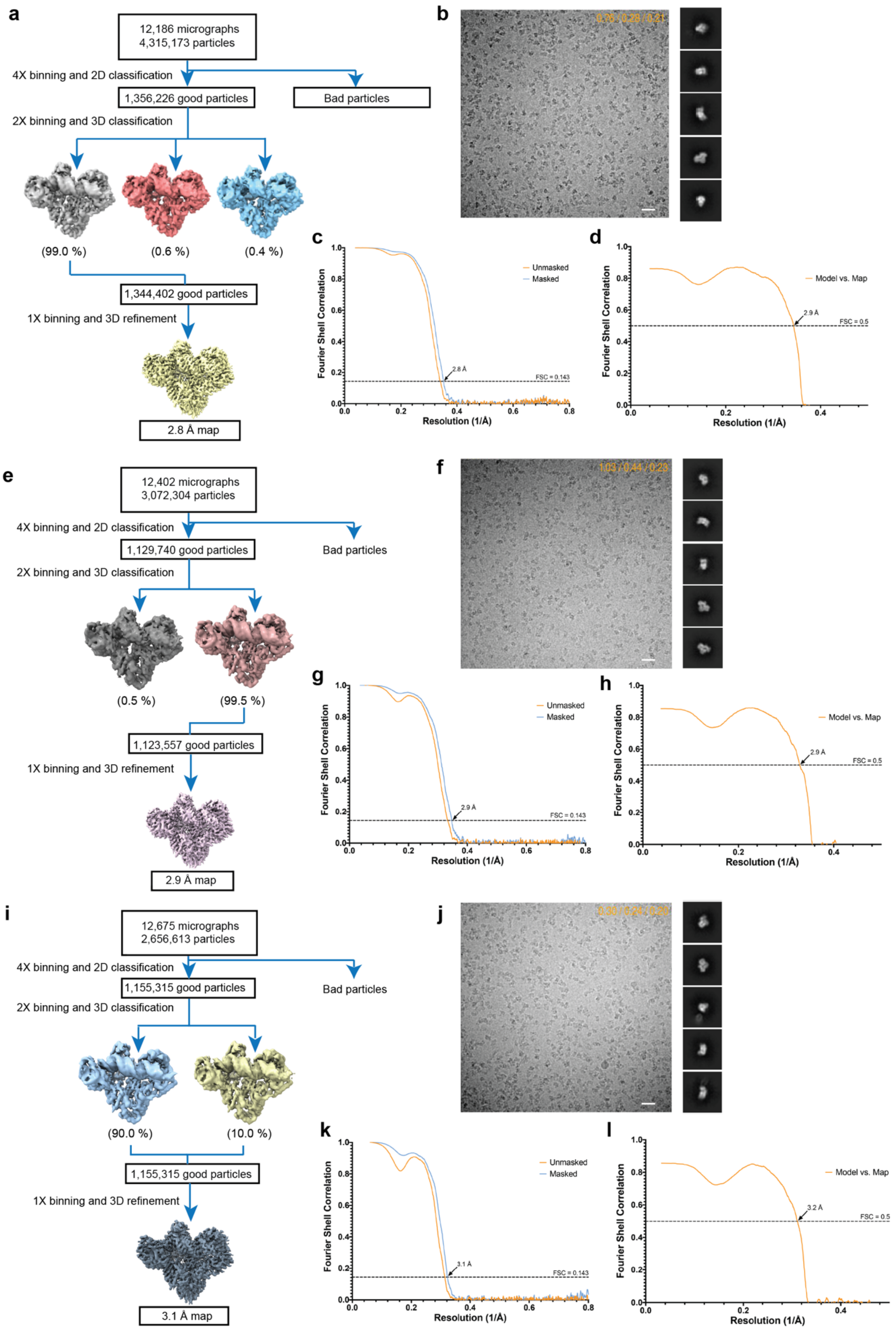
a
12,186 micrographs
4,315,173 particles
4X binning and 2D classification
1,356,226 good particles
Bad particles
2X binning and 3D classification
(99.0 %)
(0.6 %)
(0.4 %)
1,344,402 good particles
1X binning and 3D refinement
2.8 Å map
b
c
Fourier Shell Correlation
Resolution (1/Å)
Unmasked
Masked
2.8 Å
FSC = 0.143
d
Model vs. Map
2.9 Å
FSC = 0.5
e
12,402 micrographs
3,072,304 particles
4X binning and 2D classification
1,129,740 good particles
Bad particles
2X binning and 3D classification
(0.5 %)
(99.5 %)
1,123,557 good particles
1X binning and 3D refinement
2.9 Å map
f
g
Unmasked
Masked
2.9 Å
FSC = 0.143
h
Model vs. Map
2.9 Å
FSC = 0.5
i
12,675 micrographs
2,656,613 particles
4X binning and 2D classification
1,155,315 good particles
Bad particles
2X binning and 3D classification
(90.0 %)
(10.0 %)
1,155,315 good particles
1X binning and 3D refinement
3.1 Å map
j
k
Unmasked
Masked
3.1 Å
FSC = 0.143
l
Model vs. Map
3.2 Å
FSC = 0.5

Supplementary Fig. 2: Cryo-EM data processing and validation. Cryo-EM processing tree of the **(a)** apo–miniRTIC, **(e)** miniRTIC–EFZ, and **(i)** miniRTIC–NVP. Representative micrographs and 2D averages of the **(b)** apo-miniRTIC, **(f)** miniRTIC–EFZ, and **(j)** miniRTIC–NVP. Scale bar is 20 nm (white, lower right corner). Gold standard Fourier shell correlation (FSC) curve of the **(c)** apo-miniRTIC, **(g)** miniRTIC–EFZ, and **(k)** miniRTIC–NVP. The global resolution is determined by an FSC cut-off value of 0.143 (dashed line and arrow). Map-vs-model FSC curves of the **(d)** apo-miniRTIC, **(h)** miniRTIC–EFZ, and **(l)** miniRTIC–NVP. The FSC cut-off value of 0.5 is indicated by a dashed line.

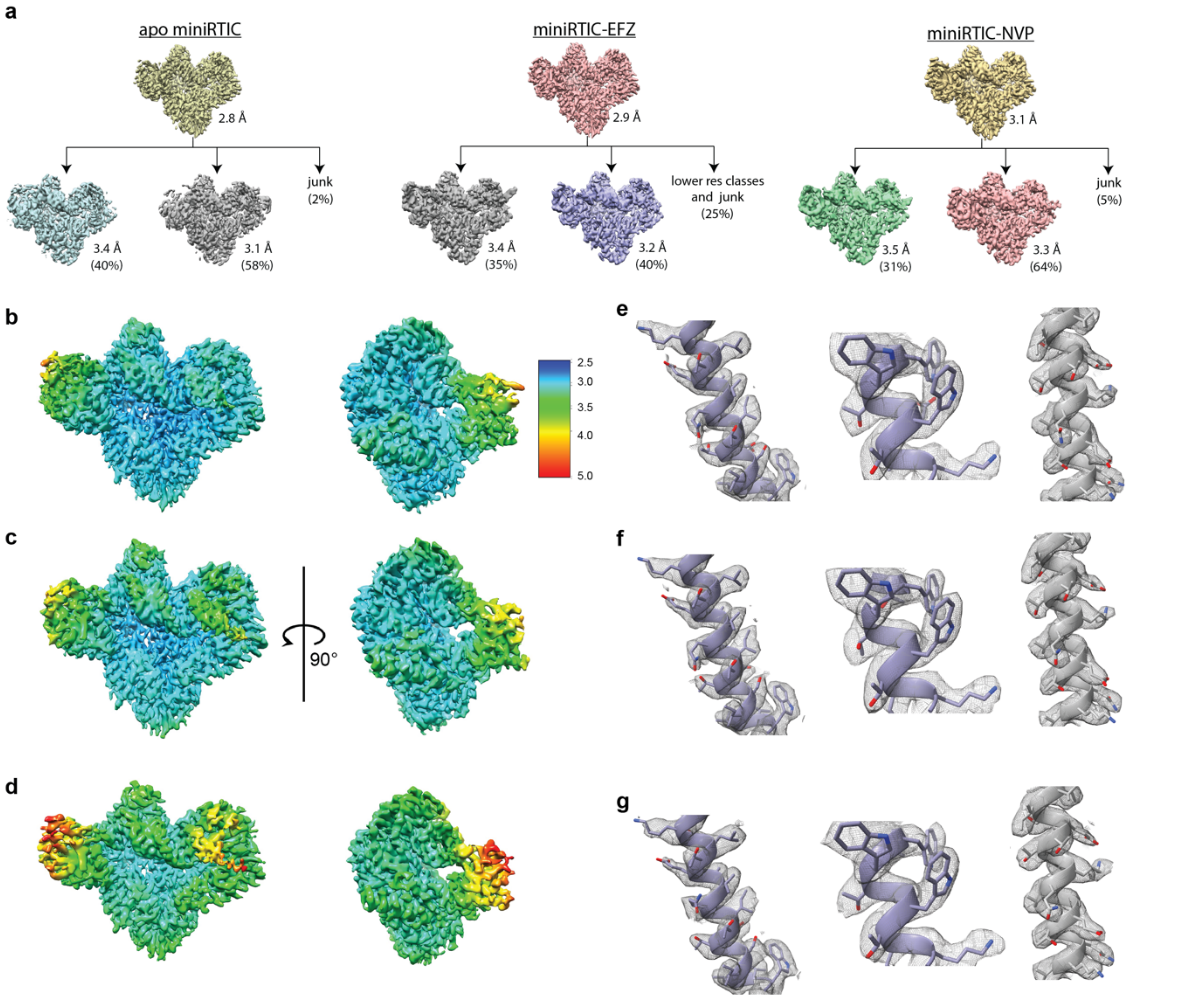

Supplementary Fig. 3: Additional cryo-EM processing, local resolution estimates, and example density. (a) Representative examples of attempts to further classify the final particle sets of all three data sets. All attempts to identify additional amounts of underlying heterogeneity did not demonstrably improve the final reconstructions in terms of resolution and quality of the density. The **(b)** apo–miniRTIC 2.8 Å, **(c)** miniRTIC–EFZ 2.9 Å and **(d)** miniRTIC–NVP 3.1 Å maps coloured according to local resolution estimated by Relion. Density and model fit for p66 subunit residues 364-383 (left), 395-403 (middle) and for p51 subunit residues 365-382 (right) for **(e)** apo–miniRTIC, **(f)** miniRTIC–EFZ and **(g)** miniRTIC–NVP.

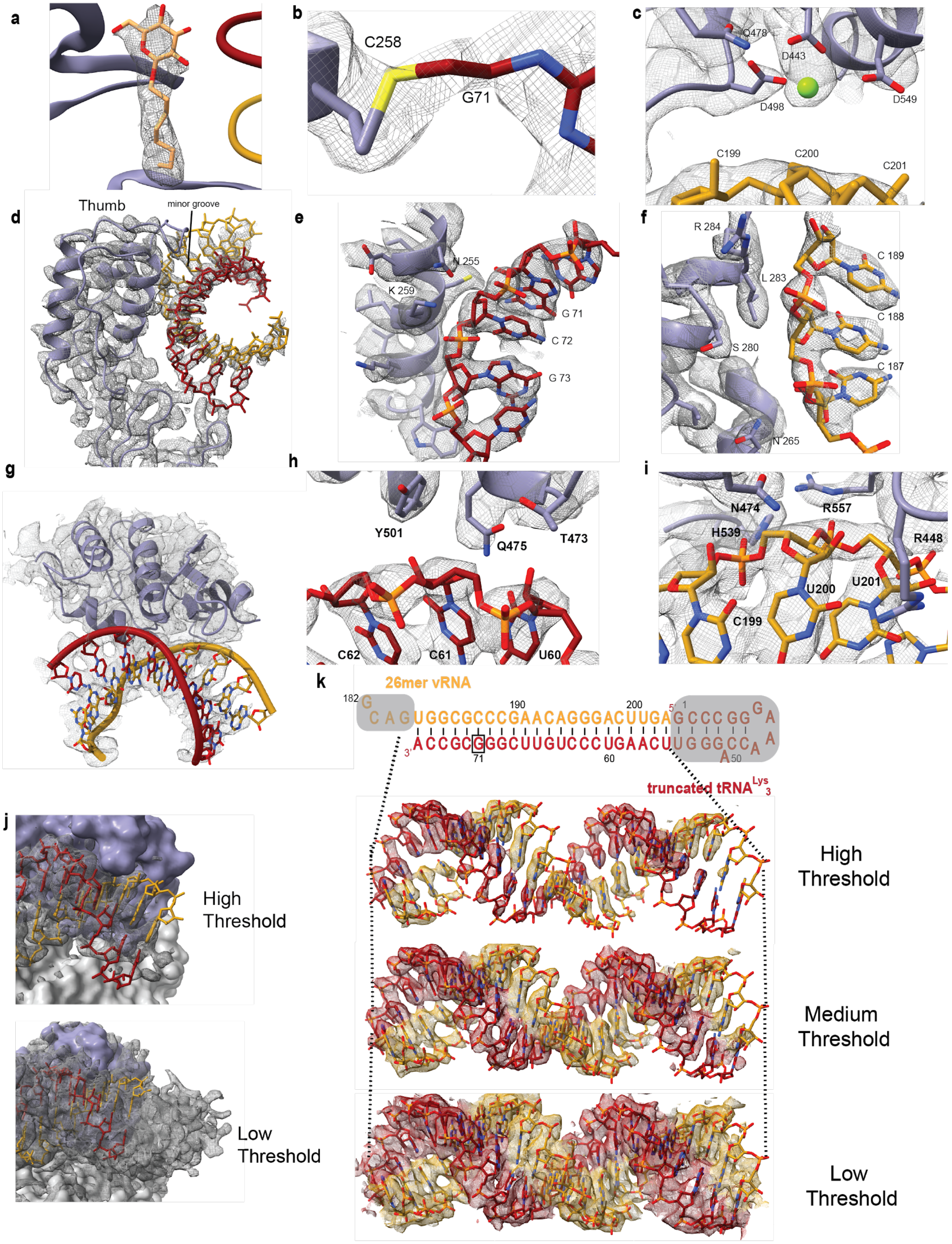

a
b
C258
G71
c
Q478
D443
D498
D549
C199
C200
C201
d
Thumb
minor groove
e
N 255
K 259
G 71
C 72
G 73
f
R 284
L 283
S 280
N 265
C 189
C 188
C 187
g
h
Y501
Q475
T473
C62
C61
U60
i
N474
R557
H539
R448
U200
U201
C199
k
26mer vRNA
182
190
200
G CAGUGGCGCCCGAACAGGGACUUGA GCCCGG GA
3' ACCGCGGGCUUGUCCCUGAACUUGGCCA A
71
60
1
50
truncated tRNA$^{Lys}_{3}$
High Threshold
Medium Threshold
Low Threshold
j
High Threshold
Low Threshold

Supplementary Fig. 4: RTIC cryo-EM density and modelling. Colouring for all panels: p66 = purple, p51 = gray, vRNA = gold, tRNA = red. **(a)** β-OG partially inserted into the NNRTI binding pocket. **(b)** Density for crosslink between C258 and modified dG71 of the tRNA primer. **(c)** RNase H active site with single bound Mg^{2+} cation. vRNA template strand (gold) is in close proximity, but not engaged. **(d)** Thumb interaction with minor groove of vRNA–tRNA. **(e)** Closeup of thumb domain α-H helix interaction with tRNA backbone. **(f)** Closeup of thumb domain α-H and α-I with vRNA backbone. **(g)** RNase H domain interaction with minor groove of vRNA–tRNA. **(h)** RNase H primer grip residues interaction with tRNA backbone **(i)** RNase H residues proximal to active site interaction with vRNA backbone. **(j)** High threshold (top) and low threshold (bottom) of density (mesh) for peripheral stem, bulged A and tetraloop density not modeled in. Density for this region is weak and could not be modeled. **(k)** Secondary structure of bimolecular RNA complex, with 26 nucleotides of vRNA (gold) annealed to 39 nucleotides of truncated $tRNA^{Lys}{}_3$ (red). Grey boxes on vRNA template overhang, tRNA stem, and tetraloop cap indicate RNA that was not modeled. Dotted lines correlate with modeled RNA nucleotides built into 2.8 Å EM map density. Map density is shown in high (top), medium, and low contour thresholds (bottom). Colouring of the RNA is the same as depicted in the secondary structure.

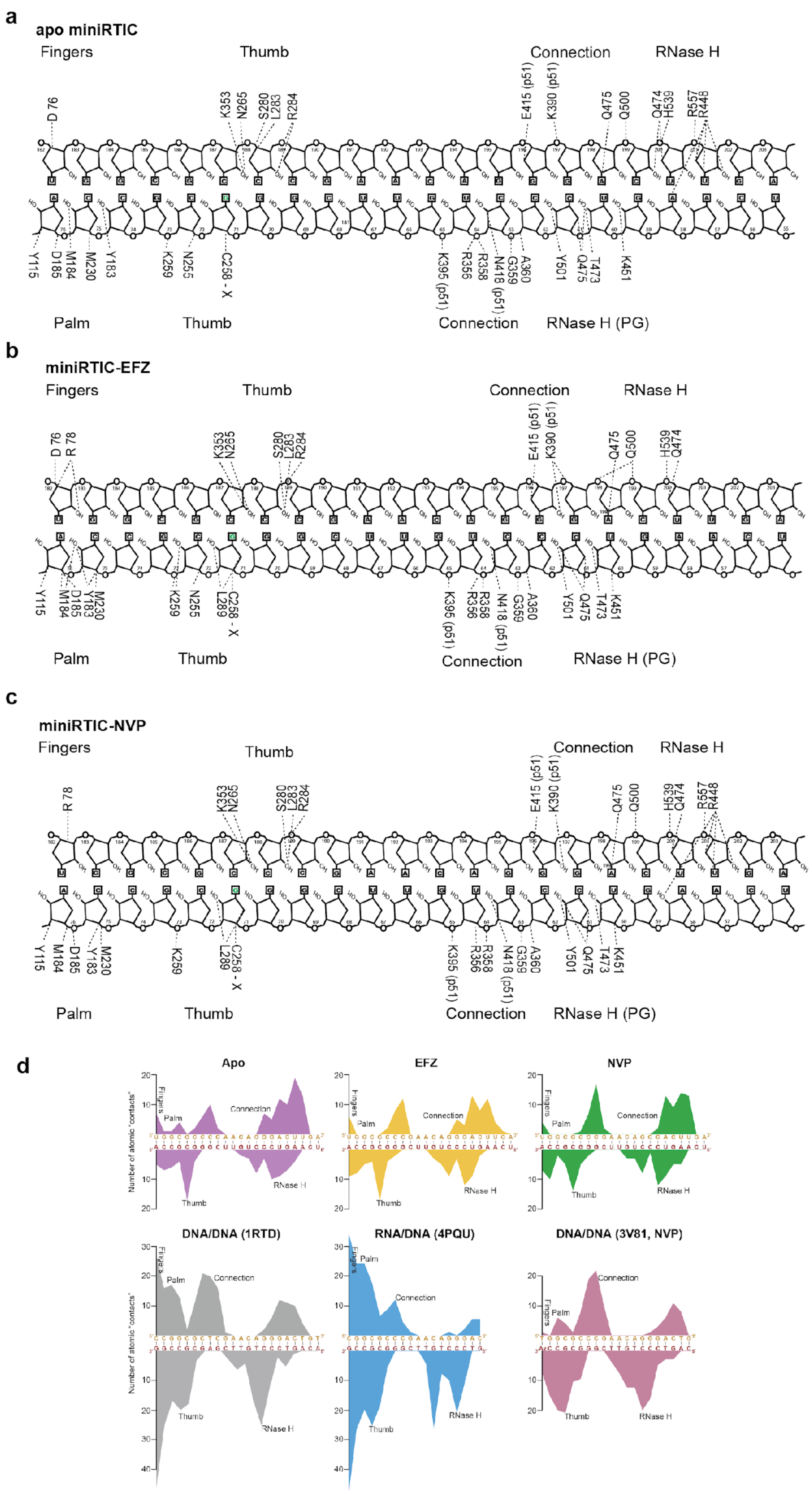
a
apo miniRTIC
Fingers
Thumb
Connection
RNase H
D 76
K353
N265
S280
L283
R284
E415 (p51)
K390 (p51)
Q475
Q500
Q474
H539
R557
R448
Y115
D185
M184
M230
Y183
K259
N255
C258 - X
K395 (p51)
R356
R358
N418 (p51)
G359
A360
Y501
Q475
T473
K451
Palm
Thumb
Connection
RNase H (PG)
b
miniRTIC-EFZ
Fingers
Thumb
Connection
RNase H
D 76
R 78
K353
N265
S280
L283
R284
E415 (p51)
K390 (p51)
Q475
Q500
H539
Q474
Y115
M184
D185
Y183
M230
K259
N255
L289
C258 - X
K395 (p51)
R356
R358
N418 (p51)
G359
A360
Y501
Q475
T473
K451
Palm
Thumb
Connection
RNase H (PG)
c
miniRTIC-NVP
Fingers
Thumb
Connection
RNase H
R 78
K353
N265
S280
L283
R284
E415 (p51)
K390 (p51)
Q475
Q500
H539
Q474
R557
R448
Y115
M184
D185
Y183
M230
K259
L289
C258 - X
K395 (p51)
R356
R358
N418 (p51)
G359
A360
Y501
Q475
T473
K451
Palm
Thumb
Connection
RNase H (PG)
d
Apo
EFZ
NVP
Fingers
Palm
Connection
Thumb
RNase H
Number of atomic "contacts"
DNA/DNA (1RTD)
RNA/DNA (4PQU)
DNA/DNA (3V81, NVP)

Supplementary Fig. 5: miniRTIC protein–RNA contact analysis. Contact map of **(a)** apo-miniRTIC, **(b)** miniRTIC–EFZ and **(c)** miniRTIC–NVP. The top strand is the vRNA template and the bottom strand is the tRNA primer. All residues, unless indicated, are located on the p66 subunit. **(d)** RT–nucleic acid contact landscape. Contacts defined as atom distances within 5 Å. Raw atom contact counts for each nucleotide on the template (top) and primer (bottom) strands are plotted on the y-axis. Apo-miniRTIC=purple, miniRTIC–EFZ=yellow, miniRTIC–NVP=green, RT–dsDNA=gray, RT–RNA–DNA=blue, RT–dsDNA–NVP=pink.

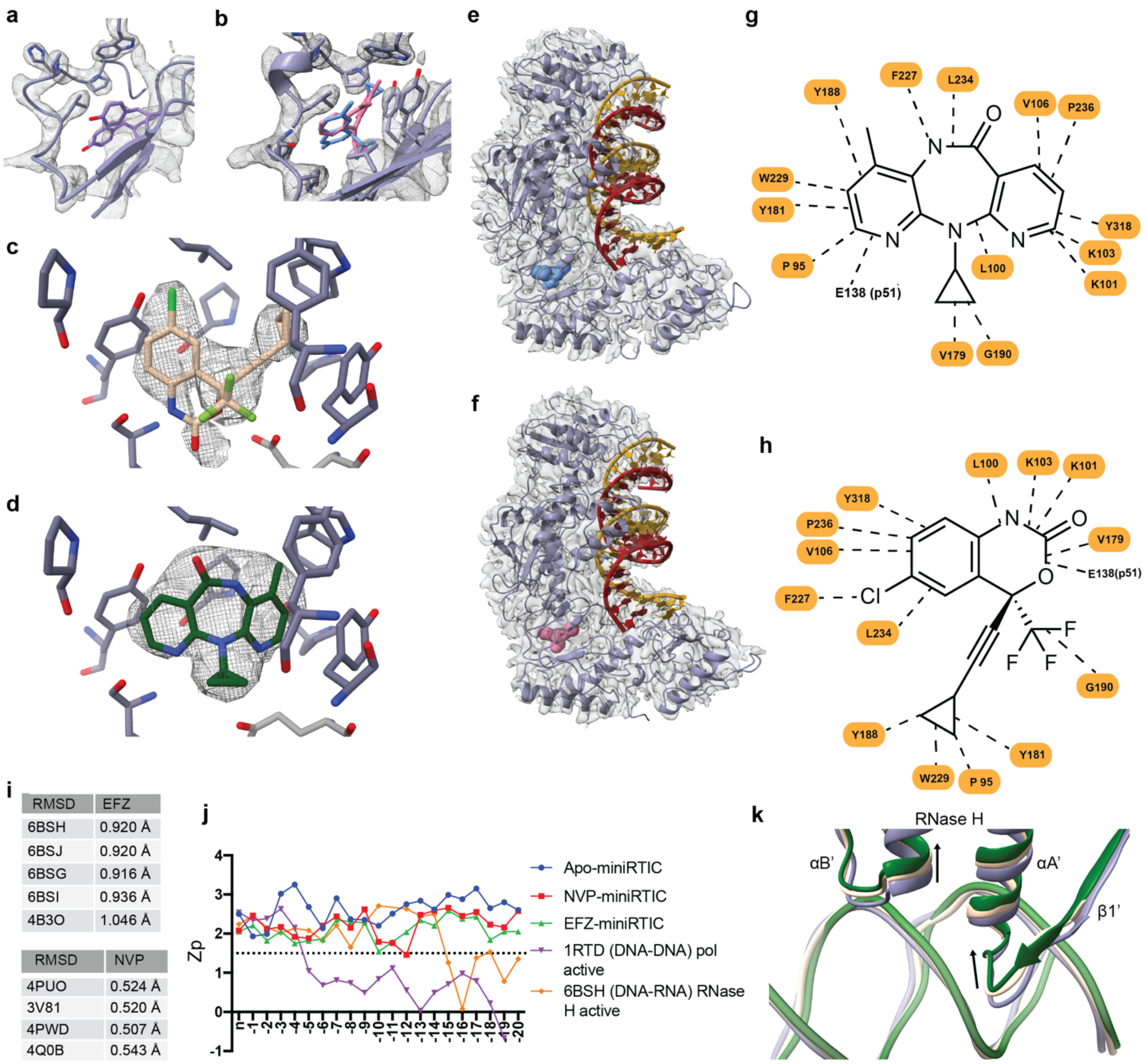

RMSD	EFZ
6BSH	0.920 Å
6BSJ	0.920 Å
6BSG	0.916 Å
6BSI	0.936 Å
4B3O	1.046 Å

RMSD	NVP
4PUO	0.524 Å
3V81	0.520 Å
4PWD	0.507 Å
4Q0B	0.543 Å

Supplementary Fig. 6: NNRTI binding to the RTIC. (a) View of apo miniRTIC binding pocket. **(b)** View of miniRTIC–NVP NNRTI binding pocket with EFZ and NVP aligned on binding pocket. Both drugs make similar interactions with RT within the binding pocket. **(c)** Front on view of EFZ (beige) and interacting residues in p66 subunit (purple) and p51 subunit (grey). **(d)** Front on view of NVP (green) and interacting residues in p66 subunit (purple) and p51 subunit (grey). **(e)** 2.9 Å map and model of miniRTIC–EFZ. EFZ is coloured in blue. **(f)** 3.1 Å map and model of miniRTIC–NVP. NVP is coloured in pink. **(g)** NVP contact map with RT residues. **(h)** EFZ contact map with RT residues. **(i)** Root mean square deviation (r.m.s.d.) of efavirenz all-atom positions bound to RTIC for structures of efavirenz bound to RT-nucleic acid structures listed by PDB ID (top); r.m.s.d. of nevirapine all-atom positions bound to RTIC nevirapine bound to RT-nucleic acid structures listed by PDB ID (bottom); r.m.s.d. values calculated after alignment on palm subdomain backbone residues. **(j)** Helical analysis using Zp data from 3DNA. Zp parameter on y-axis and base pair position on x-axis. Base pairs above the dotted line at Zp = 1.5 generally categorized

as A-like and below the line as B-like. **(k)** Compared to apo miniRTIC (purple), upon drug binding the RNase H domain shifts by ~2.0 Å in the miniRTIC–EFZ (beige) miniRTIC–NVP (green).
