# Supplementary material for: High-resolution view of HIV-1 reverse transcriptase initiation complexes and inhibition by NNRTI drugs
Source: Nat Commun. 2021 May 4;12:2500. doi: 10.1038/s41467-021-22628-9 (PMC8096811; doi:10.1038/s41467-021-22628-9)
Supplement: Supplementary file 3 — Description of Additional Supplementary Files [file 41467_2021_22628_MOESM3_ESM.pdf]

Description of Additional Supplementary Files

Supplementary Movie 1: Compaction of NNRTI binding pocket. The apical end of the primer grip (β13-β14 hairpin) shifts ~2 Å towards the base of the NNRTI binding pocket.
