# Supplementary material for: High-resolution view of HIV-1 reverse transcriptase initiation complexes and inhibition by NNRTI drugs
Source: Nat Commun. 2021 May 4;12:2500. doi: 10.1038/s41467-021-22628-9 (PMC8096811; doi:10.1038/s41467-021-22628-9)
Supplement: Supplementary file 5 — Reporting summary [file 41467_2021_22628_MOESM5_ESM.pdf]

nature research

Corresponding author(s): Elisabetta Viani Puglisi

Last updated by author(s): Mar 1, 2021

Reporting Summary

Nature Research wishes to improve the reproducibility of the work that we publish. This form provides structure for consistency and transparency in reporting. For further information on Nature Research policies, see our Editorial Policies and the Editorial Policy Checklist.

Statistics

For all statistical analyses, confirm that the following items are present in the figure legend, table legend, main text, or Methods section.

n/a	Confirmed	
☐	☒	The exact sample size (*n*) for each experimental group/condition, given as a discrete number and unit of measurement
☐	☒	A statement on whether measurements were taken from distinct samples or whether the same sample was measured repeatedly
☒	☐	The statistical test(s) used AND whether they are one- or two-sided<br>*Only common tests should be described solely by name; describe more complex techniques in the Methods section.*
☒	☐	A description of all covariates tested
☒	☐	A description of any assumptions or corrections, such as tests of normality and adjustment for multiple comparisons
☐	☒	A full description of the statistical parameters including central tendency (e.g. means) or other basic estimates (e.g. regression coefficient) AND variation (e.g. standard deviation) or associated estimates of uncertainty (e.g. confidence intervals)
☒	☐	For null hypothesis testing, the test statistic (e.g. *F*, *t*, *r*) with confidence intervals, effect sizes, degrees of freedom and *P* value noted<br>*Give P values as exact values whenever suitable.*
☒	☐	For Bayesian analysis, information on the choice of priors and Markov chain Monte Carlo settings
☒	☐	For hierarchical and complex designs, identification of the appropriate level for tests and full reporting of outcomes
☒	☐	Estimates of effect sizes (e.g. Cohen's *d*, Pearson's *r*), indicating how they were calculated

Our web collection on statistics for biologists contains articles on many of the points above.

Software and code

Policy information about availability of computer code

Data collection	SerialEM, Gatan DigitalMicrograph
Data analysis	Relion 3.0.7, cryoSPARC v2.4, GCTF v1.06, MotionCor2 v1.1, Phenix 1.18, Coot 0.8.9.3 and 0.9, UCSF Chimera 1.14, UCSF ChimeraX 1.0, Prism 8, 3DNA, Pymol 2.4, EMRinger, MolProbity, ImageQuant (GE)

For manuscripts utilizing custom algorithms or software that are central to the research but not yet described in published literature, software must be made available to editors and reviewers. We strongly encourage code deposition in a community repository (e.g. GitHub). See the Nature Research guidelines for submitting code & software for further information.

Data

Policy information about availability of data

All manuscripts must include a data availability statement. This statement should provide the following information, where applicable:

- Accession codes, unique identifiers, or web links for publicly available datasets
- A list of figures that have associated raw data
- A description of any restrictions on data availability

Cryo-EM maps of the apo-minRTIC, miniRTIC–EFZ, and miniRTIC–NVP have been deposited in the Electron Microscopy Data Bank under accession codes EMDB-22899, EMD-22900 and EMD-22901. The structure coordinates of the apo-minRTIC, miniRTIC–EFZ, and miniRTIC–NVP have been deposited in the Protein Data Bank under accession codes 7KJV, 7KJW and 7KJX. Source data are provided with the manuscript. All other data are available from the corresponding author upon reasonable request.

Field-specific reporting

Please select the one below that is the best fit for your research. If you are not sure, read the appropriate sections before making your selection.

- [x] Life sciences
- [ ] Behavioural & social sciences
- [ ] Ecological, evolutionary & environmental sciences

For a reference copy of the document with all sections, see nature.com/documents/nr-reporting-summary-flat.pdf

Life sciences study design

All studies must disclose on these points even when the disclosure is negative.

Sample size	Sample size calculations for cryo-EM dataset were not performed. Final sample size was determined when quality of final maps resolved side chain density. Biochemical assays contained sample sizes of at least n = 3 independent biological replicates, which was sufficient to obtain definitive results.
Data exclusions	Particles for cryo-EM processing were excluded from final cryo-EM reconstructions in accordance with common practices used during 2D and 3D classification.
Replication	Cryo-EM datasets contained large sample sizes with quality of final maps with resolved side chain density and therefore we did not find it necessary to reproduce this result. All biochemical purifications and assays were reproducible and performed ≥ 3 times as indicated in the manuscript.
Randomization	Randomization was not relevant to this study as the experiments, and analysis of the experimental results, required the co-authors to have knowledge of the identify of each sample.
Blinding	Binding was not relevant to the experiments performed in our study as no animal or human research subjects were involved. Blinding was not feasible as the experiments and analysis required the co-authors to have knowledge of the identify of each sample.

Reporting for specific materials, systems and methods

We require information from authors about some types of materials, experimental systems and methods used in many studies. Here, indicate whether each material, system or method listed is relevant to your study. If you are not sure if a list item applies to your research, read the appropriate section before selecting a response.

Materials & experimental systems

n/a	Involved in the study	
x		Antibodies
x		Eukaryotic cell lines
x		Palaeontology and archaeology
x		Animals and other organisms
x		Human research participants
x		Clinical data
x		Dual use research of concern

Methods

n/a	Involved in the study	
x		ChIP-seq
x		Flow cytometry
x		MRI-based neuroimaging
